# Long COVID in Children and Adolescents: A Critical Review

**DOI:** 10.3390/children11080972

**Published:** 2024-08-12

**Authors:** Maria Rothensteiner, Franziska Leeb, Florian Götzinger, Marc Tebruegge, Angela Zacharasiewicz

**Affiliations:** 1 Department of Paediatrics and Adolescent Medicine, Klinik Ottakring, 1160 Vienna, Austria; 2 Department of Paediatrics and Adolescent Medicine, Medical University Vienna, 1090 Wien, Austria; 3 Department of Paediatrics, Royal Children’s Hospital Melbourne, University of Melbourne, Melbourne, VIC 3052, Australia; 4 Department of Infection, Immunity & Inflammation, UCL Great Ormond Street Institute of Child Health, University College London, London WCN1 1EH, UK

**Keywords:** children, long COVID, PASC, post-COVID syndrome

## Abstract

(1) Background: Data on persisting symptoms after SARS-CoV-2 infection in children and adolescents are conflicting. Due to the absence of a clear pathophysiological correlate and a definitive diagnostic test, the diagnosis of Long COVID currently rests on consensus definitions only. This review aims to summarise the evidence regarding Long COVID in children and adolescents, incorporating the latest studies on this topic. (2) Methods: We designed a comprehensive search strategy to capture all relevant publications using Medline via the PubMed interface, with the initial literature search conducted in April 2023. To be included, publications had to present original data and include >50 participants with Long COVID symptoms aged between 0 and18 years. (3) Results: A total of 51 studies met the inclusion criteria, with most studies originating from Europe (n = 34; 66.7%), followed by the Americas (n = 8; 15.7%) and Asia (n = 7; 13.7%). Various study designs were employed, including retrospective, cross-sectional, prospective, or ambispective approaches. Study sizes varied significantly, with 18/51 studies having fewer than 500 participants. Many studies had methodological limitations: 23/51 (45.1%) studies did not include a control group without prior COVID-19 infection. Additionally, a considerable number of papers (33/51; 64.7%) did not include a clear definition of Long COVID. Other limitations included the lack of PCR- or serology-based confirmation of SARS-CoV-2 infection in the study group. Across different studies, there was high variability in the reported prevalence of Long COVID symptoms, ranging from 0.3% to 66.5%, with the majority of studies included in this review reporting prevalences of approximately 10–30%. Notably, the two studies with the highest prevalences also reported very high prevalences of Long COVID symptoms in the control group. There was a relatively consistent trend for Long COVID prevalence to decline substantially over time. The prevalence of Long COVID appeared to differ across different paediatric age groups, with teenagers being more commonly affected than younger children. Furthermore, data suggest that children and adolescents are less commonly affected by Long COVID compared to adults. In children and adolescents, Long COVID is associated with a very broad range of symptoms and signs affecting almost every organ system, with the respiratory, cardiovascular, and neuropsychiatric systems being most commonly affected. (4) Conclusions: The heterogeneity and limitations of published studies on Long COVID in children and adolescents complicate the interpretation of the existing data. Future studies should be rigorously designed to address unanswered questions regarding this complex disease.

## 1. Introduction

In the winter of 2019, a novel pathogenic virus, SARS-CoV-2, emerged in China and rapidly spread globally throughout 2020 [1,2]. The disease caused by this virus, closely related to the virus causing Severe Acute Respiratory Syndrome (SARS), was initially termed Coronavirus Disease 2019 (COVID-19) and is commonly referred to as simply COVID.

**Figure 1:**
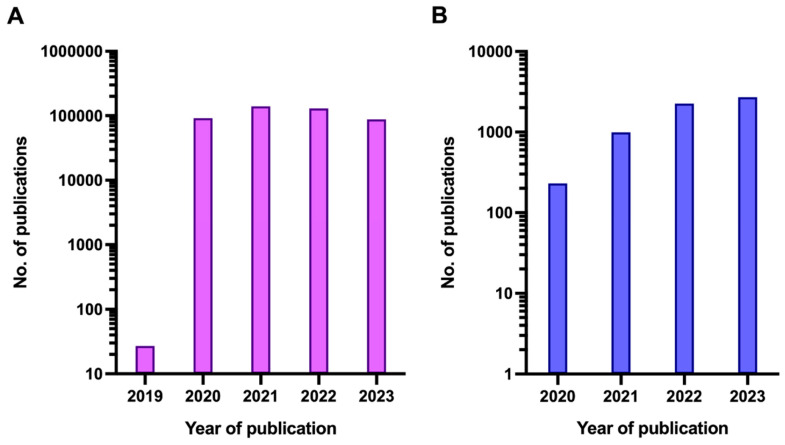
Number of publications featuring the term COVID (**A**) or any of the terms ‘Long COVID’, ‘Post-acute COVID’ or ‘PASC’ (**B**) per year since the beginning of the COVID-19 pandemic. Note that the y-axis is logarithmic.

By summer 2023, there had been more than 700 million confirmed COVID-19 cases and almost 7 million COVID-19-related deaths globally according to World Health Organisation (WHO) estimates, although it is likely that these official figures substantially underestimate the true burden of the disease and the fatalities caused by SARS-CoV-2 [3]. Simultaneous with the rise in COVID-19 cases, the literature on COVID-19 started to increase exponentially, with the number of publications peaking at a total annual rate of 138,949 in 2021 (Figure 1).

During and shortly after the first wave of the SARS-CoV-2 pandemic, two new disease entities were described: multisystem inflammatory syndrome in children (MIS-C) and Long COVID, the topic of this review. In both conditions variations in the terminology used by authors of early publications led to some degree of confusion among medical practitioners, as well as the general public. MIS-C was first described by authors from Italy and the United Kingdom [4,5], although the term was not used in either publication. In the Italian paper, ‘Kawasaki-like disease’ was used, while the UK paper referred to ‘Hyperinflammatory sepsis’. While in the United Kingdom, the term ‘Paediatric Multisystem Inflammatory Syndrome Temporally Associated with SARS-CoV-2’ (PIMS-TS) was mainly used subsequently, the term MIS-C dominated in papers originating from the United States [6,7,8]. In May 2020, the WHO adopted the latter term with some expansion [9]. 

Similar to MIS-C, a plethora of terms have been used to describe Long COVID, which will be used in this review, including long-term COVID, chronic COVID, post-acute COVID, post-COVID conditions, long-haul COVID, long-term effects of COVID, and post-acute SARS-CoV-2 infection (PASC). Recent evidence suggests that Long COVID phenotypes may cluster into four distinct phenotypes: (a) type 1: heart, kidney, and circulatory problems, (b) type 2: lung conditions, sleep disorders, and anxiety, (c) type 3: muscle pain, connective tissue disorders, and nervous system disorders, and (d) type 4: digestive and respiratory problems [10]. However, both the literature and our personal experience suggest that patients not infrequently suffer from a range of symptoms that do not conform to a single one of those phenotypes. 

Since at present there is no pathophysiological correlate of Long COVID and no diagnostic test, the diagnosis of Long COVID rests on somewhat vague definitions alone (Table 1). The WHO defined Long COVID (Post-COVID-19 condition) as “the continuation or development of new symptoms 3 months after the initial SARS-CoV-2 infection, with these symptoms lasting for at least 2 months with no other explanation” [11]. The relevant WHO fact sheet states that “Long COVID can include fatigue, shortness of breath and cognitive dysfunction; over 200 different symptoms have been reported that can have an impact on everyday functioning”. In contrast, the Department of Health and Human Services (DHHS)/Centers for Disease Control and Prevention (CDC) definition states that Long COVID is characterised by signs, symptoms, and conditions that are present four weeks or more after the initial phase of infection (Table 1) [12]. Importantly, the DHHS/CDC highlights that Long COVID is likely not a single disease entity, but rather a collection of entities.

Another definition specifically for children and adolescents has been proposed by a team of UK-based researchers, who conducted a Delphi process that included input from young patients (aged 11–17 years) suffering from Long COVID [15]. The consensus definition was as follows: “Post-COVID-19 condition occurs in young people with a history of confirmed SARS-CoV-2 infection, with one or more persisting physical symptoms for a minimum duration of 12 weeks after initial testing that cannot be explained by an alternative diagnosis” (Table 1).

While the WHO definition is probably the most commonly used definition, there is a plethora of alternative definitions frequently designed by researchers who have conducted studies on patients with Long COVID. This heterogeneity in disease definitions across studies is problematic, as individual definitions are potentially over- or under-inclusive and significantly complicate comparisons between studies, as well as data synthesis in systematic reviews. 

Several reviews on Long COVID in general and in children in particular have been published in the past [17,18,19,20,21,22,23,24]. However, even between systematic reviews (i.e., rather than narrative reviews), there is incomplete overlap in the studies included, potentially introducing bias into the data and conclusions presented in each review. It appears likely that this issue resulted from, firstly, difficulties in designing an all-encompassing search strategy that can identify all relevant papers (primarily due to the heterogeneity of terms and definitions used for Long COVID), and secondly, from the fact that all studies have been conducted in the very recent past, and consequently, the corresponding papers were at various stages of the publication process (e.g., accepted and published on journal homepage, electronically published and linked to PubMed records, or published in print) when reviews were conducted, and thereby some studies evaded detection. 

The aim of this systematic review was to produce the most comprehensive review on Long COVID in children and adolescents to date, incorporating the latest studies on this topic.

## 2. Materials and Methods

For the purpose of this review, we designed a search strategy aimed to be as inclusive as possible. After trialling a number of variations in wording and combinations, we arrived at the following search string: ((COVID) OR (SARS-CoV-2)) AND ((child) OR (adolescent) OR (paediatric) OR (paediatric)) AND ((long) OR (long-term) OR (long term) OR (prolonged) OR (post-COVID) OR (post COVID) OR (PASC) OR (persisting) OR (persistent) OR (chronic) OR (post-acute) OR (sequelae)). 

To be included in this review, publications had to fulfil the following inclusion criteria: 1. the publication reports a study on Long COVID in children and/or adolescents (aged 0–18 years) or a study including paediatric and adult patients in which the data of paediatric and adult patients can be separated; 2. the publication includes more than 50 paediatric patients with Long COVID; and 3. the publication is written in English, French, German, or Spanish. Publications not meeting all three criteria, non-peer-reviewed publications, and conference abstracts were excluded.

The initial search was conducted on 24 April 2023, using Medline via the PubMed interface. Abstracts identified in this search were subsequently screened by one of the authors (MR). In instances where it was uncertain if a publication met the inclusion criteria, a second author reviewed the abstract (MT). Following this initial round of selection, full-text articles were retrieved to determine eligibility for inclusion and to extract the relevant data in a structured fashion using a specifically designed template. All relevant publications were hand-searched for further references potentially fulfilling the inclusion criteria, as were the aforementioned reviews [17,18,19,20,21,22,23,24]. 

## 3. Results

A total of 51 articles met the inclusion criteria outlined above and were therefore included in this review. Of these, 42 studies broadly focused on Long COVID, 3 focused on mental health only, 3 on anosmia/ageusia, 2 on cardiac aspects, and 1 on pulmonary function and inflammatory markers (Table 2).

Only 2 studies were multi-country studies, while the remaining 49 studies were conducted in a single country. The large majority of studies originated from Europe (n = 34; 66.7%), followed by the Americas (n = 8; 15.7%) and Asia (n = 7; 13.7%). Among the European studies, the highest number of reports originated from the United Kingdom (n = 9), followed by studies from Italy (n = 8), Russia (n = 4), and Denmark (n = 4). Among non-European studies, a comparatively high number originated from the United States and Israel (both n = 5). The included studies employed a range of study designs and were retrospective, cross-sectional, prospective, or ambispective. Eighteen of the studies included fewer than 500 patients (Table 2); five of those included fewer than 100 patients [26,54,68,74,75]. 

### 3.1. Quality of the Included Studies and Their Limitations

As shown in Table 2, a large proportion of the included studies had methodological limitations. Twenty-three of the studies did not include a control group without prior COVID-19 infection, complicating the interpretation of the study findings. This is particularly relevant as the unusual living conditions (e.g., school closures, stay-at-home policies, and shielding at home) and social isolation during the early phases of the COVID-19 pandemic are likely to have had a substantial impact on the psychological well-being of children in general, and therefore it cannot be ruled out that many of the neuropsychiatric symptoms observed by those uncontrolled studies were the result of those conditions rather than the consequence of preceding SARS-CoV-2 infection. Importantly, studies that did include a control group without COVID-19 infection showed that the prevalence of fatigue, mood swings, anxiety, concentration difficulties, and pain symptoms in that group was substantial, commonly affecting 5–20% of the control population [29,42,52,56,61,64]. This is consistent with robust data from large-scale studies that have shown that living conditions during the pandemic adversely affected mental health, causing a significant increase in major depression and anxiety disorders [76]. Furthermore, many of the neuropsychiatric symptoms reported by various studies, such as fatigue, weakness, lack of energy, low mood, lack of concentration, sleep disturbance, and pain symptoms (e.g., headaches, myalgia, arthralgia), are highly subjective. A number of papers did not include a definition for Long COVID, complicating the interpretation of the data presented, as well as comparisons with other studies. Two studies appeared to exclusively include children who experienced symptomatic COVID-19, while children with asymptomatic infection seem to have been excluded for unclear reasons [27,28]. Other studies included children with significant pre-existing medical conditions (e.g., attention deficit hyperactivity disorder and neuropsychiatric disorders), and it remains uncertain whether certain symptoms reported, including sleep issues, concentration issues, and fatigue, were due to medical conditions that predated SARS-CoV-2 infection rather than representing Long COVID [26]. Other studies included a substantial proportion of children in whom there was no clear evidence of prior COVID-19 infection (i.e., based on PCR or serology), which potentially resulted in skewing of the reported data [27,30,71]. Furthermore, a large proportion of studies did not reliably exclude prior COVID-19 in the individuals that constituted the control group (e.g., via serological testing). None of the included studies provide a detailed analysis of the impact of prior vaccination directed against SARS-CoV-2. Finally, some of the study populations of the included studies comprised mainly adult patients, with only a minority of study subjects being in the paediatric age range [36,49,62].

### 3.2. Prevalence of Prolonged Symptoms in Children following SARS-CoV-2 Infection 

The reported prevalences of Long COVID varied widely between studies. The majority of studies included in this review reported prevalences of Long COVID following acute SARS-CoV-2 infection of approximately 10–30%. The studies that reported the lowest and highest prevalences are summarised in Table 3.

The study that observed the lowest overall prevalence (0.31%) was the study conducted by Merzon et al., which was based on electronic health records [47]. Other studies with low prevalences (4.1–5.8%) were solely based on self-reporting [27,37,48,50]. Notably, three of the five studies reporting the lowest prevalences did not include a control group [27,37,47]. The study by Molteni et al. was the only one among these that included a control group and provided the overall prevalence of Long COVID symptoms in that group [50]. The authors found that 4.4% of SARS-CoV-2-positive patients had symptoms compatible with Long COVID at 4 weeks, compared with 0.9% in the control group.

The studies reporting the highest prevalences were those by Kikkenborg Berg et al. and Stephenson et al., with prevalences of 61.9% and 66.5% in SARS-CoV-2-positive patients at >8 weeks and >12 weeks, respectively. However, both studies also reported very high prevalences of Long COVID symptoms in the control group (57.0% and 53.3%, respectively), raising the possibility that the definition of Long COVID used in the studies had a tendency to be overinclusive [41,63].

A very large study from Denmark, including more than 15,000 children who had experienced SARS-CoV-2 infection and more than 15,000 controls, found that a large proportion of participants in both groups had symptoms lasting >4 weeks (25.4% vs. 22.9%) [29]. Although this difference in proportions appears small, it was highly statistically significant. In preschool children, fatigue, loss of smell, and muscle weakness were more common in the case group than in the control group; in school children loss of smell, loss of taste, dizziness, fatigue, respiratory problems, chest pain, and muscle weakness were more common in the former than in the latter group. However, overall, the risk differences (RD) between cases and controls for each of these symptoms were small, except for loss of smell (RD 0.12), loss of taste (RD 0.10), and fatigue (RD 0.05) (Table 2).

An Italian study that included both children and adults found that persisting symptoms (>4 weeks) after SARS-CoV-2 infection were significantly more common in adults than in children [33]. At the first follow-up (1–3 months after infection), 67% of adults had persisting symptoms, compared with only 32% of children (*p* < 0.0001). Children were also more likely to have fully recovered at the 6–9 months follow-up compared with their adult counterparts (*p* = 0.01).

Another paper from Italy reported differences in the range of symptoms experienced depending on gender [34]. While headache, insomnia, and altered taste were the most common persisting symptoms in girls, persistent cough, myalgia, confusion, reduced appetite, and diarrhea predominated in boys. The same study also found that a substantial proportion of patients with initially persisting symptoms improved or showed complete resolution over time.

Several of the included studies provided data showing that the prevalence of Long COVID following SARS-CoV-2 infection differs across various paediatric age groups, with the highest prevalences observed in teenagers [37,38,42,51,53,61]. Kikkenborg Berg et al. observed persistent symptoms lasting more than 8 weeks after SARS-CoV-2 infection in 34.0% of children aged 4–11 years, compared with 42.1% of children/adolescents aged 12–14 years.

Similarly, a study analysing data from a tertiary children’s hospital in Moscow, using multivariable regression analysis, found that older age was associated with persistent symptoms lasting longer than 5 months. Compared with children <2 years of age, children aged 6–11 years and 12–18 years had a significantly higher risk of developing persistent symptoms [Odds Ratio (OR) 2.7 (1.4–5.8) and OR 2.7 (1.4–5.4)], respectively; notably, the figures in the abstract diverge from those shown in the results section of the paper] [53]. However, it has to be taken into account that determining the presence of symptoms in children younger than 2 years is challenging; notably, children of that age would not be able to report a substantial number of the symptoms included in the study’s questionnaire, such as chest tightness, dizziness, disturbed smell, loss of taste, or feeling nauseous.

Furthermore, a study from England by Atchison et al. reported that persistent symptoms occurred in 4.4% of 5–11-year-olds compared with 13.3% of 12–17-year-olds, corresponding to an adjusted odds ratio of 3.4 (95% CI 2.8–4.0) [27].

### 3.3. Range of Symptoms

The papers included in this review report a very broad range of symptoms and signs associated with Long COVID in children and adolescents. The most commonly affected systems overall were the respiratory, cardiovascular, and neuropsychiatric systems. However, the prevalence of individual symptoms in patients with Long COVID varied substantially between studies (Table 2).

A review by Lopez-Leon et al. published in 2022, which included 21 studies in a meta-analysis, found that the most common neuropsychiatric symptoms in paediatric Long COVID patients were mood changes, fatigue, and sleep disorders, followed by headaches, impaired cognition, and dizziness [20]. The most common cardiorespiratory symptoms included dyspnoea, chest pain/tightness, cough, sore throat, rhinorrhoea, and orthostatic intolerance. Other symptoms that were common included loss of taste and smell, loss of appetite, myalgia/arthralgia and hyperhidrosis. The authors also calculated the pooled Odds Ratios (OR) of 13 key symptoms using data from four studies that included both cases and controls, and found that only three of these symptoms (dyspnoea, fever, and anosmia/ageusia) were significantly more common in patients with prior microbiologically-confirmed SARS-CoV-2 infection than in controls [OR 2.69 (95% CI: 2.30–3.14), OR 2.23 (95% CI: 1.2–4.07), and OR 10.68 (95% CI: 2.48–46.03), respectively]. In contrast, there was no significant difference between cases and controls regarding mood, fatigue, headache, concentration problems, loss of appetite, rhinitis, myalgia/arthralgia, cough, sore throat, and nausea/vomiting.

A study by Sorensen et al., which evaluated persistent symptoms in PCR-confirmed SARS-CoV-2 cases (n = 2350) and SARS-CoV-2-negative controls (n = 3181) aged 15–19, years provided data stratified by gender. In females, dyspnoea [RD 2.37 (95% CI: 1.09–3.65)], chest pain [RD 2.62 (95% CI: 1.43–3.98)], dizziness [RD 2.38 (95% CI: 0.84–3.99)], fatigue/exhaustion [RD 7.37 (95% CI: 5.41–9.49)], headaches [RD 2.59 (95% CI: 0.99–4.33)], dysosmia [RD 11.77 (95% CI: 9.80–13.72)], dysgeusia [RD 9.56 (95% CI: 7.87–11.23)], reduced appetite [RD 8.89 (95% CI: 3.24–6.62)], and reduced strength in legs/arms [RD 1.75 (95% CI: 0.54–2.95)] were more common in cases than in controls. Among males, only dysosmia [RD 8.46 (95% CI: 6.08–10.72)], dysgeusia [RD 6.97 (95% CI: 5.25–8.92)], reduced appetite [RD 2.63 (95% CI: 1.10–4.28)], and reduced strength in legs/arms [RD 1.76 (95% CI: 0.61–3.03)] were observed more frequently in cases than in controls. The study also found that in both genders, difficulties concentrating, memory issues, mental exhaustion, physical exhaustion, and sleep problems occurred in a substantially higher proportion of cases compared with controls. Furthermore, the study found that cases were more likely to have received a formal diagnosis of Chronic Fatigue Syndrome and fibromyalgia compared with controls, suggesting a significant overlap between the symptoms associated with these entities and those of Long COVID [62].

A U.S. study involving children and adolescents aged 0–17 years, based on medical claims and commercial laboratory data and including 781,419 patients with COVID-19 and 2,344,257 patients without, compared the prevalence of a range of symptoms and medical conditions between the two groups [43]. The authors reported that smell and taste disturbances [adjusted hazard ratio (aHR) 1.17 (95% CI: 1.11–1.24)], circulatory signs and symptoms [aHR 1.07 (95% CI: 1.05–1.08)], malaise and fatigue [aHR 1.05 (95% CI: 1.03–1.06)], and musculoskeletal pain [aHR 1.02 (95% CI: 1.02–1.03)] were more common in cases than in controls. Surprisingly, sleep disorders [aHR 0.91 (95% CI: 0.90–0.93)], respiratory signs and symptoms [aHR 0.91 (95% CI: 0.91–0.92)], and symptoms of mental conditions [aHR 0.91 (95% CI: 0.89–0.93)] were more common in controls than in cases. Notably, all reported aHR values were close to 1, indicating that differences between cases and controls were relatively small. It was also notable that neurological conditions [aHR 0.94 (95% CI: 0.92–0.95)], anxiety and fear-related disorders [aHR 0.85 (95% CI: 0.84–0.86)] and mood disorders [aHR 0.78 (95% CI: 0.75–0.80)] were more commonly observed in controls than in cases [43].

### 3.4. Periodicity versus Chronicity of Long COVID

One survey-based study that included the parents of more than 500 children with persistent symptoms after COVID-19 examined the temporal characteristics of symptoms in children with possible Long COVID [32]. The authors found that in only 25% of those children, symptoms that had occurred during the original infection had persisted (i.e., chronicity), while 49% were experiencing an intermittent pattern of symptoms (i.e., switching between being asymptomatic and symptomatic); in a smaller proportion (19%) of participants, symptoms had newly emerged after a period of being well following COVID-19. However, one crucial limitation of that study was that in 41% of the children, the diagnosis of COVID-19 had not been confirmed by a test or a medical professional.

### 3.5. Impact of Virus Strain on the Likelihood of Long COVID

Most included papers provided no details regarding the circulating SARS-CoV-2 strains during the study period, complicating an assessment of the impact of the infecting strain on the risk of developing Long COVID. One study from Italy by Buonsenso et al. compared the prevalence of persisting symptoms after SARS-CoV-2 infection between children infected with the wild-type virus and children who had acquired infection with the Omicron variant [35]. The authors found that all symptoms included in the analyses were more common after infection with the wild-type virus (comparisons statistically significant for: fatigue, insomnia, myalgia, joint pain/swelling, and altered taste), except for persistent cough, which was more common after infection with the Omicron variant.

### 3.6. Risk Factors for Long COVID other than Virus Strain

One multicentre study that included 39 emergency departments across 8 countries found that the risk of developing Long COVID was lower in children who had PCR-confirmed COVID-19 but could be discharged from the emergency department than in children who had to be hospitalised due to the infection (4.6% vs. 9.8%) [37], indicating that greater disease severity may result in a higher chance of developing Long COVID. Although a statistical comparison of these two subgroups was not included in the original paper, we performed this comparison using data provided in the manuscript, which was statistically highly significant (Fisher’s exact test; *p* = 0.0001). The same study also found that SARS-CoV-2 positive patients who required hospitalisation had a higher risk of having persistent symptoms than matched SARS-CoV-2 negative controls that had been hospitalised during the same period. Using multiple logistic regression, the authors identified the following risk factors for Long COVID: age 14–18 years, ≥4 symptoms at ED presentation, and hospitalisation for ≥48 h.

Five of the included studies analysed whether there was an association between Long COVID and pre-existing comorbidities in children and adolescents [47,51,53,55,61]. However, four of these studies lacked a control group without exposure to SARS-CoV-2, which complicates the interpretation of the data presented [47,51,53,55]. Merzon et al. found that the risk of developing Long COVID was increased in children with attention deficit hyperkinetic disorder, chronic allergic rhinitis, and chronic urticaria. Osmanov et al. found an increased risk in children with a history of allergic disease. Pazukhina et al. observed an elevated risk in patients with neurological comorbidities or allergic respiratory disease. The only study that included a control group, by Seery et al., observed an increased risk in patients with pre-existing respiratory diseases, renal diseases, and diabetes [61].

### 3.7. Duration of Symptoms

One prospective cohort study from Italy, which included 676 participants aged 0–18 years, reported that symptoms related to presumed Long COVID tended to resolve over the course of several months [34]. In this study, patients were assessed at three different time intervals: (1) at 1–5 months (n = 355), (2) at 6–9 months (n = 157), and (3) at >12 months (n = 154). Over time, there was a significant reduction in the proportion of patients reporting myalgia (at 1st interval 10% vs. 3.2% at 3rd interval), chest pain (3.8% vs. 0), and difficulties breathing/chest tightness (3.9% vs. 0). Most other symptoms assessed also showed a decline, but the corresponding statistical comparisons did not reach significance. At the last time period, 89% of parents reported that their child had completely or almost completely recovered. Only 0.7% (equating to one family) believed that their child had shown ‘poor recovery’ at that point.

Other studies included in this review also observed a trend for Long COVID prevalence to decline over time, including the studies by Jamaica Balderas et al., Morello et al., and Pazukhino et al. [39,51,55]. The former group observed persistent symptoms in 32.6% of SARS-CoV-2 positive patients after 2 months, which declined to 9.3% after 4 and 2.3% after 6 months. Morello et al. found a prevalence of Long COVID of 23% at 3 months; of the patients who also had a follow-up at 6 months, 53% still reported symptoms, with the prevalence declining to 23% at 12 months and 19% at 18 months. However, only 77 of 294 patients with Long COVID symptoms at 3 months were followed up for the entire duration of the study [51]. Finally, Pazukhina et al. observed at least one persistent symptom after SARS-CoV-2 infection in 20% of children at 6 months, declining to 11% at 12 months [55].

### 3.8. Impact of Vaccination against SARS-CoV-2

One important issue that has been investigated after the completion of our review is whether vaccination against SARS-CoV-2 can modify the risk of developing Long COVID. Some adult studies have suggested that prior vaccination reduces the risk of Long COVID, but others have not made the same observation [77,78,79,80,81,82]. A detailed discussion of this issue is outside the scope of this paper, but a comprehensive review of the recent literature on this topic can be found elsewhere [83].

## 4. Conclusions

In conclusion, the prevalence of Long COVID in children and adolescents, as well as the range of symptoms associated with the disease and risk factors predisposing to its development, remain uncertain despite the existence of a large number of studies investigating those aspects. We found considerable variation in the reported prevalences of Long COVID in children and adolescents in the studies included in this review. Studies that included age stratification indicate that Long COVID is more common in adolescents than in younger children. The published data suggest that Long COVID can affect almost every organ system, although the respiratory, cardiovascular and neuropsychiatric systems appear to be most commonly affected. One finding consistently observed across different studies was that the prevalence of symptoms associated with Long COVID declines substantially over time, indicating that spontaneous resolution of the condition is common.

Lack of consistency between disease definitions of Long COVID considerably complicates comparisons between studies. Many existing studies had methodological limitations, including the lack of any control group or the absence of a control group in which prior COVID-19 infection had been excluded with some degree of certainty. Many studies exclusively relied on self-reporting (or parent-reporting) of symptoms and signs, rather than a detailed clinical evaluation by trained clinicians or allied health professionals.

Importantly, studies that included a control group without prior COVID-19 infection found that neuropsychiatric symptoms were common in the control population, especially in adolescents. This raises the question to what extent symptoms had been caused by SARS-CoV-2 infection and to what extent they were attributable to the unusual living circumstances during the COVID-19 pandemic. Finally, the literature on Long COVID is heavily dominated by European data, and it is unclear whether the results can be extrapolated to other geographical regions.

Further controlled studies are required to obtain more robust data on Long COVID in children and adolescents, although finding individuals who have never experienced SARS-CoV-2 infection at this point in time is likely to prove challenging. Additionally, further studies are needed to clarify the potential pathomechanisms underlying Long COVID and to identify biomarkers that could aid in the diagnosis of this condition.

## Figures and Tables

**Table 1:** Overview of key definitions for acute COVID-19 and health conditions following the acute infection.

Name of Source	Date of Publication	Definition and Key Notes
**WHO Definition of Long COVID [13]**	6 October 2021	“Post COVID-19 condition occurs in individuals with a history of probable or confirmed SARS-CoV-2 infection, usually 3 months from the onset of COVID-19 with **symptoms that last for at least 2 months** and cannot be explained by an alternative diagnosis. Common symptoms include fatigue, shortness of breath, cognitive dysfunction but also others and generally have an impact on everyday functioning. Symptoms may be new onset following initial recovery from an acute COVID-19 episode or persist from the initial illness. Symptoms may also fluctuate or relapse over time.”
**WHO Definition of Long COVID in Children and Adolescents** **[14]**	16 February 2023	“Post COVID-19 condition in children and adolescents occurs in individuals with a history of confirmed or probable SARS-CoV-2 infection, when experiencing **symptoms lasting at least 2 months which initially occurred within 3 months of acute COVID-19”.** “Current evidence suggests that symptoms more frequently reported in children and adolescents with post-COVID-19 condition compared with controls are fatigue, altered smell/anosmia and anxiety. Other symptoms have also been reported. Symptoms generally have an impact on everyday functioning such as changes in eating habits, physical activity, behaviour, academic performance, social functions (interactions with friends, peers, family) and developmental milestones. Symptoms may be new onset following initial recovery from an acute COVID-19 episode or persist from the initial illness. They may also fluctuate or relapse over time. Workup may reveal additional diagnoses, but this does not exclude the diagnosis of post COVID-19 condition. This can be applied to children of all ages, with age-specific symptoms and impact on everyday function taken into consideration”.
**Delphi research definition** **[15]**	1 April 2022	“Post-COVID-19 condition occurs in young people with a history of confirmed SARS-CoV-2 infection, with at least one persisting physical symptom for a **minimum duration of 12 weeks** after initial testing that cannot be explained by an alternative diagnosis. The symptoms have an impact on everyday functioning, may continue or develop after COVID infection, and may fluctuate or relapse over time”.
**NICE guidelines** **[16]**	First published 12/2020 Updated version 11 November 2021	**Acute COVID-19:** “Signs and symptoms of COVID-19 for up to 4 weeks”. **Ongoing symptomatic COVID-19:** “Signs and symptoms of COVID-19 from 4 weeks up to 12 weeks”. **Post-COVID-19 syndrome:** “Signs and symptoms that develop during or after an infection consistent with COVID-19, **continue for more than 12 weeks** and are not explained by an alternative diagnosis. It usually presents with clusters of symptoms, often overlapping, which can fluctuate and change over time and can affect any system in the body. Post-COVID-19 syndrome may be considered before 12 weeks while the possibility of an alternative underlying disease is also being assessed. In addition to the clinical case definitions, the term **‘long COVID’** is commonly used to describe signs and symptoms that continue or develop after acute COVID-19. It includes both ongoing symptomatic COVID-19 (from 4 to 12 weeks) and post-COVID-19 syndrome (12 weeks or more)”.
**Department of Health and Human Services (in collaboration with CDC) definition of Long COVID and PASC** **[12]**	-	“Long COVID is a patient created term broadly defined as signs, symptoms, and conditions that continue or develop after initial SARS-CoV-2 infection. The **signs, symptoms, and conditions are present four weeks or more** after the initial phase of infection; may be multisystemic; and may present with a relapsing–remitting pattern and progression or worsen over time, with the possibility of severe and life-threatening events even months or years after infection. Long COVID is not one condition. It represents many potentially overlapping entities, likely with different biological causes and different sets of risk factors and outcomes. Post-COVID-19 Conditions is equivalent to the lay term Long COVID, and is used to describe the new, returning, or ongoing health problems people can experience four or more weeks after initial infection with the SARS-CoV-2 virus, the virus that causes COVID-19. Post-acute Sequelae of SARS-CoV-2 infection is a term used in the scientific and medical communities that refers to ongoing, relapsing, or new symptoms or other health effects occurring after the acute phase of SARS-CoV-2 infection. This definition will be revised in an iterative manner based on existing and new data, medical literature, and feedback from the scientific community”. *Previous NIH definition included persistence of symptoms for longer than 30 days.*

Abbreviations: CDC = Centers for Disease Control & Prevention; NICE = National Institute for Health and Care Excellence; PASC = Post-acute Sequelae of SARS-CoV-2 infection; WHO = World Health Organization.

**Table 2:** Summary of all studies providing data on Long COVID in children and adolescents included in this review.

First Author	Study Period	Country	Number of Paediatric Patients/Type of Study Population	Study Type	Main SARS-CoV-2 Variant(s)	Definition Long COVID	Study Focus	Key Findings	Comments
**Adler 2022** **[25]**	December 2021 to January 2022	Israel	n = 1140 participants aged 5–18 years with previous SARS-CoV-2 infection n = 2092 controls without infection Online questionnaire	Cross-sectional study	Delta and Omicron variants	Symptoms >4 weeks after SARS-CoV-2 infection	Prevalence of Long COVID symptoms and associated factors	Prevalence of most symptoms was higher in children with previous infection: headaches (18.4% vs. 5.4%, *p* < 0.001), weakness (15.1% vs. 3.3%, *p* < 0.001), fatigue (12.3% vs. 6.4%, *p* < 0.001), abdominal pain (9.5% vs. 3.8%, *p* < 0.001). Symptoms were more prevalent in adolescents (12–18 years) than in children aged 5–11 years. Symptoms more frequent in participants without infection: school malfunctioning, stress, social problems, and weight changes.	Vaccination status included in analysis.
**Ashkenazi-Hoffnung 2021** **[26]**	November 2020 to April 2021	Israel	n = 90 participants ≤18 years of age who presented to a designated clinic for Long COVID	Prospective study	n.d.	NIH definition	To describe the spectrum of persisting symptoms in children with Long COVID	Most common symptoms included: fatigue (71.1%), dyspnoea (50.0%) and myalgia (45.6%). Less common symptoms included: sleep disturbances (33.3%), chest pain (31.1%), paraesthesia (28.9%), headache (28.9%), hair loss (26.7%), anosmia-ageusia or parosmia/euosmia (25.6%), gastrointestinal symptoms (20.0%), dizziness (18.9%), weight loss (18.9%), memory impairment (17.8%), vasomotor complaints (14.4%), arthralgia (14.4%), tremor (13.3%), and cough (10.0%).	No SARS-CoV-2 negative control group. Unclear if all participants had a final diagnosis of Long COVID. A substantial number of participants had pre-existing diagnoses, including asthma, ADHD, and anxiety/depressive disorders.
**Atchison 2023** **[27]**	March 2021 to March 2022	United Kingdom	n = 10,059 children and adolescents aged 5–17 years in the community (REACT-1 study)	Serial cross-sectional study	Alpha, Delta, Omicron variants	Persistent symptoms lasting ≥3 months post SARS-CoV-2 infection	Estimate the prevalence of persistent symptoms after SARS-CoV-2 infection and identify associated risk factors	≥1 persistent symptom lasting ≥3 months post infection in 4.4% (95% CI 3.7 to 5.1) of 3173 children aged 5–11 years and 13.3% (95% CI 12.5 to 14.1) of 6886 participants aged 12–17 years. Most common symptoms: age 5–11 years: persistent cough (27.4%), headaches (25.4%); age 12–17 years: loss or change of sense of smell (52.2%) and taste (40.7%) Risk factors for Long COVID: higher age and pre-existing health conditions.	No SARS-CoV-2 negative control group. Included suspected COVID-19 cases that were unconfirmed. Included only participants who previously had symptomatic SARS-CoV-2 infection.
**Bergia 2022** **[28]**	March to December 2020	Spain	n = 451 children aged <18 years with symptomatic, confirmed (PCR, antigen test, serology) SARS-CoV-2 infection n = 98 controls without history of SARS-CoV-2 infection	Retrospective study	n.d.	NICE and WHO definitions	To evaluate the prevalence of persistent symptoms after SARS-CoV-2 infection	18.4% of participants in the COVID-19 group had one or more symptoms for 4–12 weeks, 14.6% for longer than 12 weeks. Only 8.2% had 2 or more symptoms for more than 12 weeks. Odds were higher for participants ≥5 years (OR 3.0), those admitted to hospital (OR 3.9), those admitted to the PICU (OR 4.3) and those with relatives with symptoms lasting for 12 weeks or more (OR 2.8). The following symptoms were more common in the COVID-19 group than in the control group: decreased appetite, myalgia, and asthenia.	13 patients (2.9%) with MIS-C included. Included only patients who previously had symptomatic SARS-CoV-2 infection.
**Borch 2022** **[29]**	January 2020 to March 2021	Denmark	n = 15,041 children aged 0–17 years with confirmed SARS-CoV-2 infection n = 15,080 negative controls	National cohort study	n.d.	NICE guideline	To evaluate persistent symptoms after SARS-CoV-2 infection	SARS-CoV-2-positive group: 3813/15,041 (25.4%) children reported symptoms lasting >4 weeks; 1323 (34.7%) reported one symptom, 1095 (28.7%) two symptoms and 1395 (36.6%) three or more symptoms. Control group: 3446/15,080 (22.9%) children reported symptoms lasting > 4 weeks; 1870 children (54.3%) reported one symptom, 794 children (23.0%) reported two symptoms, and 782 children (22.7%) reported three or more symptoms. Despite similar proportions of participants reporting symptoms in both groups (25.4% vs. 22.9%; difference: 2.5%), this was statistically highly significant (*p* < 0.0001). SARS-CoV-2 positive preschool children suffered more often from fatigue (RD 0.05), loss of smell (RD 0.01), loss of taste (RD 0.01) and muscle weakness (RD 0.01) than controls. SARS-CoV-2 positive school children suffered more often from loss of smell (RD 0.12), loss of taste (RD 0.10), fatigue (RD 0.05), respiratory problems (RD 0.03), dizziness (RD 0.02), muscle weakness (RD 0.02) and chest pain (RD 0.01) than controls.	
**Brackel 2021** **[30]**	December 2020 to February 2021	The Nether-lands	n = 89 children suspected of having Long COVID aged 2–18 years, reported by local paediatricians based at various hospitals across the country	Cross-sectional observational study	n.d.	Not provided	To determine the number of paediatric patients who have been referred to a specialist by a family doctor and are experiencing Long COVID symptoms	The most common complaints were fatigue (87%), dyspnoea (55%), concentration difficulties (45%) and headaches (38%). 36% of children had severe limitations in their daily activities.	In a substantial proportion of patients, SARS-CoV-2 infection was not microbiologically confirmed.
**Buonsenso 2021** **[31]**	March to October 2020	Italy	n = 109 patients aged <18 years, at least 30 days after being diagnosed with SARS-CoV-2 infection (PCR confirmed) (on average 162.5 ± 113.7 days after infection) Questionnaire-based Included both patients with symptomatic and asymptomatic SARS-CoV-2 infection	Cross-sectional study	n.d.	Not provided	To evaluate persistent symptoms after SARS-CoV-2 infection in paediatric patients	Overall, 41.8% of participants completely recovered, 35.7% reported 1–2 symptoms, 22.5% had more than 3 symptoms. 27.1% of participants (35 of 68 children) reported at least one persistent symptom for ≥120 days. Most common symptoms: Insomnia (18.6%), respiratory problems (14.7%), nasal congestion (12.4%), fatigue (10.8%), muscle (10.1%) and joint pain (6.9%), and impaired concentration (10.1%).	2.3% of participants had MIS-C. Alternative diagnoses were not ruled out. No SARS-CoV-2 negative control group.
**Buonsenso 2022** **[32]**	January 2020 to January 2021	Mainly United Kingdom and USA	n = 510 participants aged 1–18 years Questionnaire-based survey (parent-led)	Prospective survey	n.d.	Symptoms for >4 weeks after COVID-19	To evaluate prolonged symptoms after SARS-CoV-2 infection based on parental perception	Mean time of persistent symptoms after SARS-CoV-2: 8.2 months (standard deviation: 3.9) Most common symptoms: Tiredness and weakness (87.1%), fatigue (80.4%), headache (78.6%), abdominal pain (75.9%), muscle aches (68.4%), muscle and joint pain (60.6%), post-exertional malaise (53.7%), rash (52.4%), unexplained irritability (51.4%), dizziness (48.0%). Most common changes since SARS-CoV-2 infection: changes concerning energy levels (83.3%), mood (58.8%), sleep (56.3%), appetite (49.6%), lower levels of activity (90%), concentration difficulties (60.6%), memory problems (45.9%), difficulty doing everyday tasks (40%).	In 41% of participants COVID-19 had not been confirmed by a test or medical professional. No SARS-CoV-2 negative control group.
**Buonsenso 2022** **[33]** *	April 2020 to April 2021	Italy	Larger cohort (n = 507) which included n = 179 children and n = 101 adults with PCR-confirmed SARS-CoV-2 infection, who had at least one follow-up at 1–3 months after infection n = 37 SARS-CoV-2 negative controls (children) n = 49 SARS-CoV-2 negative controls (adults) Assessment via phone call and face-to-face visits Follow-ups at 1–3 months and at 6–9 months	Prospective cohort study	n.d.	Symptoms for >4 weeks after COVID-19	To describe and compare long-term symptoms after SARS-CoV-2 infection in adults and children living in the same households	A significantly higher number of adults reported at least one persistent symptom after COVID-19 at first follow-up compared with children (67%, 68/101 vs. 32%, 57/179; *p* < 0.001). Participants aged ≤18 years had a higher probability of full recovery in comparison to adults at both follow-ups at 1–3 months (*p* = 0.001) and 6–9 months (*p* = 0.01). 16.7% of children experienced co-existing symptoms from ≥ 2 symptom categories at 1–3 months follow-up, 3.3% reported symptoms from ≥ 3 categories. At 6–9 months follow-up, 5.1% of children reported symptoms from ≥ 2 and 1.4% from ≥ 3 categories.	ISARIC Global COVID-19 follow-up protocol was used as a screening tool for persistent symptoms. Additional data were kindly provided by one of the authors
**Buonsenso 2022** **[34]**	April 2020 to April 2022	Italy	n = 679 PCR-confirmed participants aged 0–18 years Follow-up at 1–5, 6–9, and ≥12 months post-SARS-CoV-2 diagnosis Assessment via phone call, a survey, and face-to-face visits	Prospective cohort study	Wild type virus (488/679 participants), Alpha, Delta, Omicron variants	Delphi research definition Symptoms present for at least 4 weeks since the diagnosis of SARS-CoV-2 infection	To assess the prevalence of persistent symptoms after SARS-CoV-2 and to evaluate risk factors, including virus variants	Most frequent persistent symptoms: fatigue (19%), headache (12%), insomnia (7.5%), muscle pain (6.9%), and confusion with concentration issues (6.8%). Overall improvement over time was reported: at the 1–5 months follow-up, 4% of parents reported ‘poor recovery’; 1.3% at 6–9 months; and 0.7% at ≥12 months follow-up. Patients not recovered by 6–9 months had a lower probability of recovery during the subsequent follow-up period. In total, 86% of participants described a full or almost complete recovery.	ISARIC Global COVID-19 follow-up protocol for children was used. Symptom patterns according to virus variants were analysed. No SARS-CoV-2 negative control group.
**Buonsenso 2023** **[35]**	n.d.	Italy	n = 1243 participants, median age 7.3 years (4.0–10.2). Follow-up at 3, 6, 12 months (18 months for pre-Omicron infections)	Prospective study	Omicron (70.8%) Delta (19.9%), Alpha (6%), and wild-type (3.3%) variants	Symptoms for >3 months after acute COVID-19	To evaluate the risk of persistent symptoms depending on SARS-CoV-2 variant	Infection with pre-Omicron variants was associated with a significantly higher rate of persistent symptoms at 3 and 6 months. Highest odds ratios (ORs) were observed with the initial variants: wild-type SARS-CoV-2: 4.1 (2.2–7.7, *p* < 0.001) at 3 months and 8.6 (4.3–17.1, *p* < 0.001) at 6 months; Alpha variant: OR 4.4 (2.7–7.1, *p* < 0.001) at 3 months and 9.9 (5.8–16.8, *p* < 0.001) at 6 months.	
**Chevinsky 2021** **[36]** *	March to June 2020	United States	n = 305 inpatient patients aged <18 years n = 305 matched controls n = 2368 outpatient patients n = 2368 matched controls Healthcare database-based analysis	Matched cohort study	n.d.	‘Late conditions’ defined as conditions not previously recorded as underlying or acute COVID-19 conditions	To assess the type, association, and timing (1–4 months) of post-COVID conditions	Children who previously had COVID-19 were not at a higher risk of experiencing post-COVID conditions compared with children who did not have COVID-19.	Analyses heavily focused on adult data. Study did not assess persisting symptoms (i.e., symptoms that started with COVID-19 and continued).
**Funk 2022** **[37]**	March 2020 to January 2021	8 countries	n = 1884 PCR-confirmed SARS-CoV-2–positive children aged <18 years (1802 not hospitalised, 564 hospitalised) n = 1701 SARS-CoV-2–negative controls Follow-up 90–120 days after COVID-19 Participant recruitment in 39 paediatric emergency departments in 8 countries	Prospective cohort study	n.d.	Persistent, new or returning symptoms >90 days after SARS-CoV-2 infection	To evaluate the prevalence of Post COVID-19 condition 90 days after an emergency department visit	Overall, 5.8% (95% CI, 4.8–7.0%) of SARS-CoV-2 positive participants had persistent symptoms. 9.8% (95% CI 7.4–13.0%) of hospitalised children vs. 4.6% (95% CI, 3.6–5.8%) of non-hospitalised children had persistent symptoms. Most common symptoms in the SARS-CoV-2–positive group: respiratory (e.g., cough, difficulty breathing, or shortness of breath; 2.0%) and systemic (e.g., fatigue or weakness; 1.8%). In the negative matched control group, 5.0% of hospitalised children and 2.7% of non-hospitalised children reported prolonged symptoms. Risk factors for PCC: Hospitalisation for ≥48 h, ≥4 acute symptoms, age ≥14 years.	Comparison of hospitalised vs. non-hospitalised cases not provided in manuscript—calculated using data provided in manuscript: Fisher’s exact test *p* = 0.0001. No SARS-CoV-2 negative control group.
**Guido 2022** **[38]**	February to November 2021	Italy	n = 322 participants after SARS-CoV-2 infection aged between 1.5 and 17 years Evaluation at disease onset, after 1 month, and after 3–5 months	Prospective study	n.d.	Persistent symptoms for more than 3 months after onset	To evaluate the presence of neurological symptoms and psychological effects	22% of participants had persistent symptoms at 3–5 months. Most prevalent neurological symptoms: headache (7.5%), fatigue (6.8%), and anosmia (2.2%). Fewer symptoms were found in the group of 1.5–5-year-old patients compared with the 6–17-year-old patients. Other persistent symptoms: Cognitive, behavioural and mood problems (14%), sleep disturbances (13%), and changes in eating habits (10%).	No SARS-CoV-2 negative control group. Unclear how SARS-CoV-2 infection was determined.
**Jamaica Balderas 2023** **[39]**	July 2020 to December 2021	Mexico	n = 215 patients <18 years with positive SARS-CoV-2 PCR test and/or IgG test Follow-up at 2, 4, 6, and 12 months	Prospective study	n.d.	Persistent symptoms >12 weeks after acute SARS-CoV-2 infection	To describe clinical experience with Long COVID patients in a paediatric centre	Persistent symptoms were observed in 32.6% of patients after 2, 9.3% after 4, and 2.3% after 6 months. Main persistent symptoms: dyspnoea, dry cough, runny nose and fatigue.	8.4% of participants had MIS-C. No SARS-CoV-2 negative control group.
**Katsuta 2023** **[40]**	February 2020 to April 2022	Japan	n = 1697 SARS-CoV-2-positive patients <16 years	Prospective study	pre-Delta, Delta, Omicron	Symptoms >28 days after SARS-CoV-2 infection	To evaluate persisting symptoms after SARS-CoV-2 infection	Prolonged symptoms were reported in 3.2% (n = 55). Common symptoms were dysosmia (1.1%), dysgeusia (1.0%), fever (0.8%), fatigue (0.7%), and cough (0.5%). Persistent symptoms were more likely to occur in patients who had fever or dysosmia at acute presentation.	No SARS-CoV-2 negative control group.
**Kikkenborg Berg 2022** **[41]**	January 2020 to July 2021	Denmark	n = 6630 patients aged 15–18 years with confirmed SARS-CoV-2 infection n = 21,640 controls Questionnaire-based Paediatric Quality of Life (PedsQL) Children’s Somatic Symptoms Inventory-24 (CSSI-24)	National cross-sectional study	n.d.	Persistent symptoms >2 months after SARS-CoV-2 infection	To evaluate quality of life, psychological and social well-being, school absence during the pandemic, and long-term symptoms	Cases had higher odds of experiencing at least one Long COVID symptom lasting ≥2 months compared with the control group [3159 (61.9%) vs. 12,340 (57.0%), odds ratio 1.22 (95% CI 1.15–1.30); *p* < 0.0001]. Symptoms significantly more common in cases than controls: chest pain, headache, sore throat, dizziness, loss of appetite, trouble breathing, palpitations, and cough. Cases had better quality of life scores on PedsQL, as well as significantly higher physical, emotional, social, and school functioning mean scores. Cases also reported lower symptom scores in CSSI-24.	
**Kikkenborg Berg 2022** **[42]**	January 2020 to July 2021	Denmark	n = 10,997 SARS-CoV-2-positive children aged 0–14 years n = 33,016 negative controls Questionnaire-based	Cross-sectional study	Alpha, Delta variants	Persistent symptoms for >8 weeks after the positive SARS-CoV-2 test	To examine the prevalence, severity, and duration of persistent symptoms, as well as impact on quality of life, number of missed school or daycare days, and psychological and social effects after SARS-CoV-2 infection	In all age groups, cases had higher odds of experiencing at least one persistent symptom (for more than 3 months) than controls: 0–3 years age group (435 [36.4%] of 1194 vs. 872 [22.6%] of 3855; OR 1.94 [1.68–2.23], *p* < 0.0001) 4–11 years age group (1710 [34.0%] of 5023 vs. 5356 [29.2%] of 18,372; OR 1,28 [1.19–1.37], *p* < 0.0001) 12–14 years age group (1204 [42.1%] of 2857 vs. 3966 [36.8%] of 10,789; 1.26 [1.11–1.32], *p* < 0.0001). Symptoms significantly more prevalent in the case group included: fatigue, headache, dizziness, sore throat, muscle/joint pain, chest pain, trouble breathing, cough, and loss of appetite. There was a trend towards better quality-of-life scores in emotional and social functioning for cases compared with controls in older children.	Similar rates of pre-existing co-morbidities in cases and controls.
**Kompaniyets 2022** **[43]**	March 2020 to January 2022	United States	n = 781,419 patients aged 0–17 years with SARS-CoV-2 infection n = 2,344,257 negative controls aged 0–17 years Analysis based on a large medical claims database Symptoms were classified by International Classification of Diseases, Tenth Revision, Clinical Modification (ICD-10-CM)	Retrospective study	n.d.	Symptoms >4 weeks after SARS-CoV-2 infection	To assess persistent symptoms after SARS-CoV-2 infection	Patients with SARS-CoV-2 infection were less likely to experience the following symptoms/conditions: Respiratory symptoms (aHR = 0.91), sleeping disorders (0.91), neurological conditions (0.94), anxiety-related conditions (0.85), mood disorders (0.78), and muscle disorder (0.94). Patients with SARS-CoV-2 infection were more likely to experience the following symptoms/conditions: Smell and taste disturbances (aHR = 1.17), circulatory symptoms (1.07), malaise and fatigue (1.05), musculoskeletal pain (1.02), pulmonary embolism (2.01), myocarditis and cardiomyopathy (1.99), venous thromboembolic event (1.87), renal failure (1.32), type 1 diabetes (1.23), coagulation and haemorrhagic disorders (1.18), type 2 diabetes (1.17), and cardiac dysrhythmias (1.16).	
**Kuitunen 2022** **[44]**	January 2021 to September 2022	Finland	n = 132 visits to general practitioners coded with a diagnosis of Long COVID in children aged 1–14 years. control group: 21,633 visits of controls aged 15–64 years aged for Long COVID.	Retrospective register-based study	n.d.	not available	To analyse the incidence of formally-diagnosed Long COVID cases in Finnish children	The visit rate due to Long COVID was very low in children compared to adults: age 1–6: 7.9 per 100,000 visits, age 7–14: 19.0 per 100,000 visits, age 15–64: 541.6 per 100,000 visits.	Lack of clinical data.
**Lorman 2023** **[45]**	March 2020 to June 2022	United States	n = 14,399 patients <21 years, divided into 3 cohorts: n = 1309 post-acute Sequelae of SARS-CoV-2 (PASC) cases (using ICD-10 diagnosis U09.9) n = 6545 children with SARS-CoV-2 infection (PCR, antigen, or serology positive) n = 6545 children without SARS-CoV-2 infection	Cohort study	Pre- and post-Omicron variants	NIH definition of PASC Persistent symptoms for >30 days after SARS-CoV-2 infection	To identify clusters of PASC-associated diagnoses	Significant increase was observed in multiple symptoms related to cardiovascular, respiratory, neurological, psychological, endocrine, gastrointestinal, and musculoskeletal systems among children with PASC. Typical symptoms in PASC cases (compared with both control groups) included dyspnoea, abnormalities in breathing, and malaise/fatigue.	Study is based on Electronic Health Records from 9 different hospitals. n = 3806 (26.4%) patients were aged 16–20 years, including n = 346 PASC cases, n = 1730 SARS-CoV-2 negative, and n = 1730 SARS-CoV-2 positive patients.
**Messiah 2022** **[46]**	March 2021 to January 2022	United States	n = 312 children aged 0–19 years diagnosed with COVID-19 (n = 286) or MIS-C (n = 26)	Retrospective study	n.d.	NICE criteria	To assess the presence of acute (<30 days) and chronic (≥30, 60–120, and >120 days) long-term COVID symptoms	26.9% of children with MIS-C and 15.3% without MIS-C reported persistent symptoms for ≥30 days. Females were almost twice as likely to report persistent symptoms compared to males.	No SARS-CoV-2 negative control group. Electronic Health Records based study. Additional data were kindly provided by one of the authors.
**Merzon 2022** **[47]**	February 2020 to June 2021	Israel	n = 20,601 patients aged 5–18 years after SARS-CoV-2 infection (PCR tested), including n = 65 patients with Long COVID Based on an online computerised Israeli database of patient demographics, medical visits, laboratory tests, hospitalisations, and prescriptions	Population-based cross-sectional study	n.d.	Persistent symptoms ≥12 weeks after SARS-CoV-2 infection	To identify demographic, clinical, and socioeconomic factors associated with Long COVID	65 of 20,601 participants (0.32%) had a diagnosis of Long COVID Variables associated with Long COVID: hospitalisation due to COVID-19 infection (aOR 44.7), recurrent acute SARS-CoV-2 infection(s) within 180 days (aOR 43.7), symptomatic COVID-19 infection (aOR = 5.3), older age (aOR 1.4) Pre-existing conditions associated with Long COVID: ADHD (aOR 2.0), chronic allergic rhinitis (aOR = 2.7) and chronic urticaria (aOR 8.1).	Small number of participants with Long COVID. No SARS-CoV-2 negative control group. Only included patients with formal diagnosis of Long COVID. Additional data were kindly provided by one of the authors.
**Miller 2022** **[48]**	June 2020 to May 2021	United Kingdom	n = 1062 patients ≤17 years with past or present evidence of SARS-CoV-2 infection n = 3970 patients without evidence of SARS-CoV-2 infection Questionnaire-based with weekly and monthly surveys	Household-based community cohort study	n.d.	NICE guidelines Persistent symptoms lasting > 4 weeks	To evaluate the prevalence of persistent symptoms after SARS-CoV-2 infection	43 of 1062 (4.1%; 95% CI, 2.9–5.4%) patients with previous SARS-CoV-2 infection reported persistent symptoms. The prevalence of persisting symptoms in the whole cohort (including patients with or without evidence of SARS-CoV-2 infection) was 2.6% (129/5032 patients; 95% CI, 2.1–3.0%).	Not all patients without evidence of SARS-CoV-2 infection had undergone laboratory testing.
**Mizrahi** 2023 [49] *	March 2020 to October 2021	Israel	n = 118,308 SARS-CoV-2 positive patients ≤18 years n = 118,308 negative controls Electronic medical records from an Israeli nationwide healthcare organisation	Retrospective nationwide cohort study	Wild-type, Alpha, Delta variants	Divided into early (30–180 days) and late (180–360 days) time periods after infection Defined as prolonged or new symptoms >4 weeks after acute onset	To determine clinical sequelae of Long COVID during the first year after mild SARS-CoV-2 infection	Elevated risk according to age group: Age 0–4: Conjunctivitis (HR 1.18, 1.08–1.29) and dyspnoea (HR 1.22, 1.11–1.35) only during the early phase Age 5–11: Conjunctivitis (HR 1.24, 1.07–1.43) only during the early phase. Sore throat (HR 1.54, 1.20–1.97) only during the late phase Age 12–18 Anosmia and dysgeusia (HR 23.5, 5.48–100.86), dyspnoea (HR 1.7, 1.36–2.12) and weakness (HR 1.66, 1.41–1.96) only during the early phase In the entire cohort (including adults), there was a trend for anosmia/dysgeusia, dyspnoea, weakness, chest pain, and palpitations to decline over time.	Only patients with mild disease. Study included additional adult cases and controls. Anosmia and dysgeusia were grouped together in the analysis.
**Molteni 2021** **[50]**	September 2020 to January 2021	United Kingdom	n = 1734 children with positive SARS-CoV-2 test aged 5–17 years n = 1734 negative SARS-CoV-2 matched controls	Prospective cohort study	n.d.	NICE guidelines	To evaluate illness duration and symptoms of COVID-19 in children	77 (4.4%) of 1734 children had symptoms for ≥ 28 days with low symptom burden (median 2 symptoms, IQR 1–4) in comparison to the first week of illness (median 6 symptoms, IQR 4–8). Most common persisting symptoms were fatigue, headache, and anosmia. 25 (1.8%) of 1379 children reported symptoms for ≥ 56 days. The most common symptoms in those 25 children over their entire illness were anosmia (84.0%), headache (80.0%), sore throat (80.0%), and fatigue (76.0%). Few children among the negative controls (0.9%) had symptoms for at least 28 days.	
**Morello 2023** **[51]**	February 2020 to October 2022	Italy	n = 1243 patients aged 0–18 years after SARS-CoV-2 infection In-clinic follow-up at a post-COVID clinic at 3, 6, 12 and 18 months after onset	Prospective cohort study	Wild-type virus, Alpha, Delta, Omicron variants The majority had infection with Omicron (70.8%).	Delphi definition	To evaluate risk factors for Long COVID and recovery rates	23% (294/1243) met the criteria for Long COVID at 3 months. Of 268 patients that had Long COVID at the 3-month and follow-up at 6 months 143 (53%) reported symptoms. At 12 months 38/167 (23%) and at 18 months 15/77 (19%) reported symptoms. Most common symptoms at 3 months: Fatigue (55.1%), exertional dyspnoea (26.2%), headache (23.5%) and gastrointestinal symptoms (19.0%), muscle pain (18.0%), concentration/memory problems (10.5%), joint pain (10.5%), and chest pain (9.9%). Risk factors for Long COVID: >10 years of age (OR 1.2; CI 1.2–1.3), presence of comorbidities (OR 1.7; CI 1.1–2.5) and hospitalisation during the acute phase (OR 4.8; CI 1.9–12.1). Reduced risk for Long COVID: infection with Omicron variant (OR 0.6; CI 0.5–0.8) and being asymptomatic during the acute phase of the infection (OR 0.5; CI 0.2–0.9).	No SARS-CoV-2 negative control group. 89% of participants had only mild infection. Additional data were kindly provided by one of the authors
**Nugawela 2022** **[52]**	January to March 2021	United Kingdom	n = 3246 participants aged 11–17 years after SARS-CoV-2 infection n = 3893 negative matched controls	Questionnaire-based cohort study	n.d.	Delphi definition	To create a predictive model for Long COVID symptoms in children and young people	3 months after PCR test 25.2% (817/3246) of SARS-CoV-2 positive patients and 18.5% (719/3893) of the control group experienced ≥ 1 impairing ongoing symptoms (*p* < 0.001; calculated based on data provided in the manuscript) A risk prediction equation to identify those at highest risk of Long COVID 3 months after SARS-CoV-2 infection was established.	Data from the children and young people with Long COVID (CLoCk) study was used.
**Osmanov 2022** **[53]**	April to August 2020	Russia	n = 518 hospitalised patients ≤18 years after PCR-confirmed SARS-CoV-2 infection Median follow-up time since hospital discharge: 256 days	Cross-sectional cohort study	Wild-type virus	Persistent symptoms >5 months	To evaluate long-term outcomes and to identify risk factors in children previously hospitalised after SARS-CoV-2 infection	126 patients (24.3%) experienced persistent symptoms at follow-up. 44 patients (8.4%) reported symptoms in ≥2 categories. Most common symptoms: Fatigue (n = 53, 10.7%), sleep disorders (n = 36, 6.9%), and sensory problems (n = 29, 5.6%). Risk factors for Long COVID: Older age, with 6–11 years (OR 2.7 (95% CI 1.4 to 5.8) and 12–18 years (OR 2.7, 95% CI 1.4 to 5.4) and history of allergic diseases (OR 1.7, 95% CI 1.04 to 2.7).	ISARIC Global follow-up protocol was used. Only hospitalised patients included. No SARS-CoV-2 negative control group.
**Palacios 2022** **[54]**	February to December 2021	United States	n = 82 adolescents with an average age of 15.2 years Evaluation at median time of 3.5 months after SARS-CoV-2 infection, 49% had a follow-up 2–3 months later	Single- center, retrospective cohort study	n.d.	Persistent symptoms for more than 4 weeks after infection	To evaluate persistent pulmonary abnormalities after COVID-19	80% of participants reported ≥2 symptoms (cough, chest pain, dyspnoea at rest or during exercise), 67% of participants followed up at about 6.5 months post-infection had persisting exertional dyspnoea. 77% of participants had normal spirometry, 17% (n = 14) had obstructive deficits, and 6.1% (n = 5) had restrictive deficits. Normal results were shown on plethysmography or diffusion capacity.	No SARS-CoV-2 negative control group.
**Pazukhina 2022** **[55]**	April to August 2020	Russia	n = 360 previously hospitalised children (median age 9.5 years) with confirmed SARS-CoV-2 infection Two follow-up telephone interviews at 6 and 12 months after discharge	Prospective cohort study	n.d.	WHO definition	To assess prevalence and risk factors of Post Covid-19 Condition	At least one persistent symptom was present in 20% (95% CI 16–24) of children at 6 months, decreasing to 11% (95% CI 8–14) at 12 months. Most common symptoms at 6 months: Fatigue 9%, dermatological 5%, neurocognitive 4% and sleep-related symptoms 4%. At 12 months: decrease of symptoms to 4%, 2%, 2%, and 1% was observed, respectively. Risk factors associated with post COVID condition: neurological comorbidities both at 6 months (OR 4.4, 1.4 to 15.7) and 12 months (OR 9.0 2.6 to 34.8), allergic respiratory diseases at 12 months (OR 2.7, 1.04 to 6.5).	No SARS-CoV-2 negative control group.
**Pinto Pereira 2023** **[56]**	September 2020 to March 2021	England	n = 6407 SARS-CoV-2 positive patients aged 11–17 years n = 6542 SARS-CoV-2 negative controls Questionnaire-based study 6 months after acute illness	National cohort study	n.d.	Delphi definition	To evaluate physical and mental health 6 months after SARS-CoV-2 infection	24.5% of SARS-CoV-2 positive patients and 17.8% of negative controls had Long COVID symptoms at 6 months after infection. Common symptoms in both groups: Tiredness, shortness of breath, and headaches.	
**Pinto Pereira 2023** **[57]**	September 2020 to March 2021	England	n = 2909 SARS-CoV-2 positive patients aged 11–17 years n = 2177 SARS-CoV-2 negative controls Questionnaire based follow-up after 6 and 12 months	Prospective study	Wild-type, Alpha (B.1.1.7) variants	Delphi research definition	To assess health and well-being 6 and 12 months after SARS-CoV-2 infection	Prevalence of Long COVID at 6 and 12 months: SARS-CoV-2 positive group: 6 m: 748/2909 (25.7%) 12 m: 785/2909 (27.0%). SARS-CoV-2 negative group: 6 m: 362/2177 = (16.6%) 12 m: 458/2177 = (21.0%).	Additional data were kindly provided by the first author.
**Roessler** 2022 [58] *	January 2019 to December 2020	Germany	n = 11,950 patients aged 0–17 years with COVID-19 n = 59,750 matched control group Mean follow-up time: 236 days after acute illness	Retrospective matched cohort study based on health insurance data	Pre-Omicron variants	WHO definition, but symptoms >3 months after diagnosis of COVID-19	To evaluate morbidity after COVID-19 in children and adolescents	Incidence Rate Ratios (IRR) of documented health-related problems was significantly higher in the COVID-19 group (IRR: 1.3, 95% CI: 1.25-1.35, *p* < 0.01). Specific outcomes with the highest IRR were malaise/fatigue/exhaustion (IRR 2.3), cough (IRR 1.7), throat/chest pain (1.7).	Data from German statutory health insurance organisations.
**Roge 2021** **[59]**	July 2020 to April 2021	Latvia	n = 236 patients with COVID-19 aged 1 month to 18 years n = 142 SARS-CoV-2 negative controls with other community-acquired Infections	Ambi-directional cohort study	n.d.	NICE criteria	To evaluate ongoing symptoms 1–6 months after SARS-CoV-2 infection Median follow-up time after COVID-19: 73.5 days (IQR 43–110 days), 69 days in the control group (IQR 58–84 days)	44.5% (n = 105) of SARS-CoV2 positive patients reported persistent symptoms 12 weeks after infection. Most common symptoms: irritability (27.6%, n = 29), changes in mood (26.7%, n = 28), and fatigue (19.2%, n = 20). In comparison to the control group, persistent symptoms such as fever [adjusted Odds Ratio (ORa) 4.0, 95% CI: 1.4–11.6; *p* = 0.01], fatigue (ORa 8.7, 95% CI: 2.5–29.9; *p* = 0.001), rhinorrhea (ORa 2.6, 95% CI: 1.3–5.4; *p* = 0.008) anosmia/dysgeusia (ORa 11.2, 95% CI: 1.4–89.1; *p* = 0.02), headaches (ORa 12.9, 95% CI: 1.7–99.6; *p* = 0.01), nocturnal sweating (ORa 16.7, 95% CI: 2.1–130.4; *p* = 0.007) were significantly associated with SARS-CoV-2 infection. Cognitive difficulties (concentration, memory, attention, mood changes, irritability and anxiety/depression) were also significantly more common in cases than controls.	Control group consisted of patients with other community-aquired infections.
**Sakurada 2023** **[60]** *	February 2021 to October 2022	Japan	n = 54 patients diagnosed with Long COVID aged 11–18 years n = 398 patients diagnosed with Long COVID aged >19 years	Retrospective observational study	Alpha, Delta, Omicron variants	Symptoms >4 weeks after onset of SARS-CoV-2 infection	To evaluate clinical characteristics of Long COVID in teenagers compared with adults	In both groups the most frequent complaints were fatigue (teenagers: 55.6% vs. adults: 61.1%; *p* = 0.44) and headache (35.2% vs. 21.9%; *p* = 0.03). Other common symptoms included dyosmia, dysgeusia, insomnia, dyspnoea and hair loss, but no significant differences between teenagers and adults were detected.	No SARS-CoV-2 negative control group.
**Seery 2023** **[61]**	June 2020 to June 2021	Argentina	n = 639 patients 1–17 years old with confirmed SARS-CoV-2 infection n = 577 SARS-CoV-2 negative controls Parent questionnaire 6 months after testing	Observational study	Pre-Omicron variants	WHO criteria Symptoms >3 months after SARS-CoV-2 infection	To evaluate long-term symptoms of SARS-CoV-2 and associated risk factors	Significantly more patients with previous infection had at least one symptom ≥3 months compared with the control group (34% vs. 13%, *p* < 0.0001). There was a 3 to 7-fold increased risk of headache, dizziness, loss of taste, dyspnoea, cough, fatigue, muscle pain and weight loss in the subgroup that had experienced SARS-CoV-2 infection. Risk factors for Long COVID were older age, comorbidities and symptomatic infection.	
**Sorensen 2022** **[62]**	September 2020 to April 2021	Denmark	PCR confirmed SARS-CoV-2 cases aged 15–19 years: ♀ n = 1512 ♂ n = 838 SARS-CoV-2 negative controls: ♀ n = 2163 ♂ n = 1018	Prospective questionnaire -based study	Wild type, Alpha	WHO definition	To evaluate the risk differences between SARS-CoV-2 positive participants and negative controls for persistent symptoms 6–12 months after SARS-CoV-2 infection	There was no risk difference between cases and controls regarding PTSD, anxiety, or depression. There was an increased risk difference in cases regarding chronic fatigue (in ♀ and ♂) and fibromyalgia (in ♀). Dysosmia, dygeusia, reduced appetite, and reduced strength were more common in cases than in controls (in ♀ and ♂), dyspnoe, chest pain, dizziness, fatigue and headache were more common in cases than in controls in ♀ only. Difficulties concentrating (♀ 33% vs. 9.6%, RD 25.3; ♂ 17.1% vs. 5.1%, RD 13.4), memory issues (♀ 26% vs. 5.5%, RD 22.6; ♂ 13.7% vs. 3.6%, RD 11.3), mental exhaustion (♀ 46.8% vs. 18.9%, RD 29.3; ♂ 24.3% vs. 9.8%, RD 15.8), physical exhaustion (♀ 46.4% vs. 12.2%, RD 36.3; ♂ 23.2% vs. 6.5%, RD 37.9), and sleeping problems (♀ 23.9% vs. 11.3%, RD 13.6; ♂ 15.6% vs. 7.1%, RD 9.2) were more common in cases than in controls.	Figures based on supplementary data file of the original manuscript and additional data kindly provided by the author.
**Stephenson 2022** **[63]**	January to March 2021	United Kingdom	n = 3065 SARS-CoV-2 positive adolescents aged 11–17 years n = 3739 matched negative controls	Longitudinal cohort study	Mainly Alpha	Persistent symptoms for more than 3 months after SARS-CoV-2 infection	To evaluate the incidence and clinical phenotype of Long COVID	At 3 months 2038 (66.5%) positive participants and 1993 (53.3%) negative controls had any symptoms, and 928 (30.3%) from the positive group and 603 (16.2%) from the control group had three or more symptoms. At 3 months the most common symptoms in the SARS-CoV-2 positive group vs. the control group were: tiredness (39.0% vs. 24.4%), shortness of breath (23.4% vs. 10.4%) and headaches (23.2% vs. 14.2%). All three comparisons were statistically highly significant (*p* < 0.0001).	
**Stephenson 2023** **[64]**	January to March 2021	United Kingdom	n = 1658 SARS-CoV-2 positive 11–17 year old patients (PCR confirmed) n = 1737 SARS-CoV-2 negative controls Follow-up online questionnaire 3 and 6 months after PCR test	Prospective study	n.d.	No clear definition provided	Evaluation of ongoing symptoms after SARS-CoV-2 infection	There were 35.9% SARS-CoV-2 patients with at least 1 symptom at baseline, compared with 67.8% at 3 and 56.6% at 6 months. In the control group 9.2% had at least 1 symptom at baseline, 53.3% at 3 and 35.3% at 6 months. The 11 most common symptoms (>10%) in SARS-CoV-2 patients were: Fever, chills, headache, loss of smell, muscle pain, persistent cough, sore throat, skipping meals, shortness of breath, tiredness, and dizziness. After 3 months, prevalence of those symptoms was reduced and a smaller decline was observed after 6 months. The same symptoms and trends were observed among the control group at lower prevalence. New symptoms were reported after 6 months in both groups.	Data from national matched cohort study (The CLoCk).
**Valenzuela 2022** **[65]**	March 2020 to December 2021	Chile	SARS-CoV-2 positive (PCR-tested) hospitalised patients at 3 tertiary hospitals n = 216 hospitalised patients n = 67 (31%) of those were obese	Observational study	Alpha, Gamma, Lambda and Delta variants	Symptoms >4 weeks after onset of symptoms	Association of obesity and post-acute sequelae of SARS-CoV-2 infection	At the 6 months follow-up obesity was associated with dyspnoea (aOR 9.91, 95% CI 1.92–51.10) and muscle weakness (aOR 20.04, 95% CI 2.50–160.65). There was a significant association between obesity and muscle weakness in all age groups, but the association between obesity and dyspnoea was only significant in patients < 12 years.	Obesity was associated with ICU admission, oxygen requirement, non-invasive ventilatory support, superinfections and bacterial pneumonia.
**Warren-Gash 2023** **[66]**	November 2021 to April 2022	United Kingdom	n = 7797 children (SARS-CoV-2 positive and negative) Data from COVID-19 School Infection Survey (SIS) 3 survey rounds at 173 schools Round 1: Nov.–Dec. 2021 Round 2: Jan.–Feb. 2022 Round 3: Mar.–Apr. 2022 Questionnaire-based in round 1 and 3	Prospective observational study	Pre- and during Omicron	Delphi research definition Symptoms lasting >12 weeks after onset of SARS-CoV-2 infection	To assess the prevalence of Long COVID comparing persisting symptoms between SARS-CoV-2 positive and negative children	Symptoms that were significantly more common in SARS-CoV-2 positive patients than the control group comprised: 4–11 years: loss of smell/taste and cardiovascular 11–16 years: loss of smell/taste, cardiovascular and systemic symptoms (fever, chills, weakness or tiredness) 16–18 years: loss of smell/taste and pulmonary.	Control group comprised of children without a history of SARS-CoV-2 or without known positive test result; prior infection not excluded via serology.
**Pulmonary function/inflammatory markers**									
**Sommen** 2023 [67] *	December 2020 to May 2021	Norway	n = 405 SARS-CoV-2 positive participants (aged 12–25 years; median: 18 years) n = 111 negative controls Baseline and follow-up at 6 months after mild SARS-CoV-2 infection	Prospective study	Alpha (B1.1.7.) variant	WHO definition	Investigation of immunological blood markers and pulmonary function after SARS-CoV-2 infection	Elevated levels of chemokines/cytokines (eotaxin, MCP-1, and IP-10) were detected 6 months after mild SARS-CoV-2 infection. No difference between patients with Long COVID and those without in any of the immunological parameters investigated. No difference in pulmonary function between the SARS-CoV-2 positive and negative groups regardless of long-lasting symptoms.	Only included patients with mild SARS-CoV-2. Study included adults; no age breakdown provided.
**Long COVID and mental health**									
**Akçay 2022** **[68]**	April 2020 to February 2021	Turkey	n = 74 patients aged 11–17 years after SARS-CoV-2 infection Revised Children’s Anxiety and Depression Scale (RCADS) and UCLA-loneliness scale were used. Baseline inflammatory markers at acute phase of infection	Retrospective study	n.d.	Not provided	To investigate how baseline inflammation levels, internalising symptoms, and feelings of loneliness are related in SARS-CoV-2 survivors	Only a weak association between baseline C-reactive protein levels and major depressive disorder was found (r = 0.258, *p* = 0.027). Higher levels of anxiety in the patient or the parent were associated with a higher risk of developing depression, as was loneliness in the patient.	No SARS-CoV-2 negative control group.
**Blankenburg 2022** **[69]**	March to April 2021	Germany	n = 188 seropositive students (median age 15 years) n = 1365 seronegative controls	Cross-sectional survey (school-based)	n.d.	Persisting symptom >12 weeks after acute SARS-CoV-2 infection	Assessing certain neurocognitive, pain, and mood symptoms	High rates of neurocognitive, pain and mood symptoms were observed in both groups. Female students reported a consistently higher prevalence of neurocognitive, pain and mood symptoms. Only one statistically significant difference was detected: seropositive students felt less sadness (*p* < 0.05).	
**Shachar-Lavie 2023** **[70]**	November 2020 to August 2021	Israel	n = 103 patients with Long COVID (serologically or PCR confirmed) n = 113 uninfected healthy children aged 4–18 years in the control group Parent questionnaire-based and structured interviews	Case–control study	n.d.	Symptoms >4 weeks after SARS-CoV-2 infection	To examine the consequences of Long COVID on children’s mental health	In Long COVID patients, there was a significantly higher prevalence of memory difficulties. No group differences were found in other functional aspects (connection with friends and engagement in physical activities), concentration impairment, as well as emotional and behavioural problems.	
**Cardiac impact**									
**Erol 2021** **[71]**	March to June 2021	Turkey	n = 121 SARS-CoV-2 positive patients aged 0–18 years (positive PCR or positive history) n = 95 controls without known contact with COVID-19 cases Evaluation of blood pressure, electrocardiography, and echocardiography results	n.d.	n.d.	No clear definition of Long COVID Included patients that had SARS-CoV-2 infection >1 month and <1 year ago	To evaluate persistent symptoms after SARS-CoV-2 infection in relation to cardiovascular health	37.2% (n = 45) of SARS-CoV-2 positive patients had persistent symptoms during the study period. The most common persistent symptoms were dizziness ± syncope (15.6%) and palpitations ± chest pain (11.1%). chest and back ache (51.1%). Significant differences between the study and control group were found in systolic blood pressure values and echocardiographic parameters (left ventricular ejection fraction, relative wall thickness, and tricuspid annular plane systolic excursion). No significant differences in heart rates in ECG, PR intervals, and QTc values between both groups.	Not all cases were microbiologically confirmed. Prior SARS-CoV-2 infection in controls not excluded via serology. Only the blood pressure values of patients >2 years were evaluated.
**Sabatino 2022** **[72]**	February 2020 to December 2021	Italy	n = 157 SARS-CoV-2 positive patients aged 0–18 years (microbiologically confirmed) n = 107 healthy patients in the control group Three follow-up groups: <180 days, 180–240 days, >240 days Performing of transthoracic echocardiogram and speckle-tracking echocardiography	Single-centre observational prospective study	n.d.	No definition of Long COVID provided	To evaluate cardiac mechanics in previously healthy patients after asymptomatic or mild SARS-CoV-2 infection	A significant reduction in left ventricular global longitudinal strain in SARS-CoV-2 group compared to controls (SARS-CoV-2: −20.5 ± 2.9%; controls: −21.8 ± 1.7%; *p* < 0.001). No significant differences between the three follow-up groups were identified.	Only patients with asymptomatic or mild disease (WHO stages 0 or 1) were included. No association between virus variants and cardiac findings.
**Anosmia/Ageusia**									
**Mariani 2023** **[73]**	Beginning of the pandemic until October 2022	Italy	n = 1250 patients aged <19 years referred to an outpatient clinic after microbiologically confirmed SARS-CoV-2 infection Follow-up at 3, 6, 12, and 18 months after infection	Retrospective study	Wild type, Alpha, Delta, and Omicron variants	Symptoms for >12 weeks after confirmed SARS-CoV-2 infection that cannot be explained otherwise	To evaluate risk factors for persistent anosmia and dysgeusia after SARS-CoV-2 infection Also, to evaluate SARS-CoV-2 variants, hospitalisation, vaccination, and olfactory/gustative dysfunction during the acute phase of infection	During the acute phase of infection, 5.1% of all patients had anosmia and 4.2% had dysgeusia. At 3 months (n = 1250) 1% had anosmia; 1% dysgeusia At 6 months (n = 1224) 0.6% had anosmia; 0.6% dysgeusia At 12 months (n = 181) 1.7% had anosmia; 1.1% dysgeusia At 18 months (n = 87) 2 (2.3%) had anosmia; only 1 (1.1%) dysgeusia. Risk factors for persistent anosmia and dysgeusia were onset during during the acute phase of SARS-CoV-2, and Infection/illness due to a pre-Omicron variant.	Impact of virus variants and vaccination status included in analysis. No significant association between vaccination status and persistence of dysgeusia and anosmia. Onlyfew patients were assessed at 18 months.
**Namazova-Baranova 2022** **[74]**	n.d.	Russia	n = 61 patients aged 6–18 years after SARS-CoV-2 infection n = 20 controls without previous SARS-CoV-2 infection Examination at 6–8 weeks after recovery and after 1 year Olfactory and gustatory sensitivity test scores were used, the three-component olfactory test and a parent questionnaire	Prospective study	n.d.	Not provided	To evaluate smell and taste after SARS-CoV-2 infection	Compared with controls, a reduction in the ability to smell and taste was observed in the SARS-CoV-2 group. Olfactory sensitivity in participants who had recovered from SARS-CoV-2 returned to normal levels after 6–12 months.	Small sample size; very small cohort at 1 year (n=21). Control group was also small. Previous SARS-CoV-2 infection not excluded in control group (e.g. by serology).
**Rusetsky 2021** **[75]**	April and May 2020	Russia	n = 79 hospitalised patients aged 6–17 years with PCR-confirmed SARS-CoV-2 infection Follow-up at day 5 and day 60 Evaluation based on the SNOT-22 standardised questionnaire, psychophysical olfactory test, and telephone survey	Prospective cross-sectional study	n.d.	Not provided	To evaluate the olfactory status after acute SARS-CoV-2 infection	At day 0 (D0) 54/79 (68.4%) participants had olfactory impairment, which declined to 41/79 (51.9%) at D5. At D60 all participants had fully recovered (n = 72; 7 lost to follow-up). In 3 patients, recovery only occurred after D30.	Small sample size. No SARS-CoV-2 negative control group. Patients with a “severe need for oxygen support” were excluded.

* Also includes adult patients; Abbreviations: ADHD = attention deficit hyperactivity disorder; adjusted hazard ratio = aHR, adjusted odds ratio = aOR, D = day; n.d. = no data; MIS-C = Multisystem Inflammatory Syndrome in Children; NIHR = UK National Institute for Health Research; PASC = post-acute sequelae of SARS-CoV-2 infection; RD = risk difference; WHO = World Health Organization.

**Table 3:** Summary of studies reporting the lowest (top) and highest (bottom) prevalence figures of Long COVID in children and adolescents, including how the diagnosis was established.

Author	Year	Duration of Symptoms (weeks)	Self-Assessment	Medical Review	Electronic Health Records	SARS-CoV-2 Positive Patients (No.)	Long COVID (%)	Negative Controls (No.)	Long COVID (%)	Difference
Merzon et al. [47]	2022	>12			●	20,601	0.31% with formal diagnosis	No SARS-CoV-2 negative control group		
Miller et al. [48]	2022	>4	●			1062	4.1%	3970	No data	No data
Atchison et al. [27]	2023	>12	●			10,059	4.4%	No SARS-CoV-2 negative control group		
Molteni et al. [50]	2021	>4	●			1734	4.4%	1734	0.9%	3.5%
Funk et al. [37]	2022	>12	●			1884	5.8%	No SARS-CoV-2 negative control group		
Kikkenborg Berg et al. [42]	2022	>8	●			10,997 4–11 years: 5023 12–14 years: 2857	4–11 years: 34.0% 12–14 years: 42.1%	33,016 4–11 years 18,372 12–14 years: 10,789	4–11 years: 29.2% 12–14 years: 36.8%	4–11 years: 4.8% 12–14 years: 5.3%
Roge et al. [59]	2021	>12		●		236	44.5%	142	No data	No data
Kikkenborg Berg et al. [41]	2022	>8	●			6630	61.9%	21,640	57.0%	4.9%
Stephenson et al. [15]	2022	>12	●			3065	66.5%	3739	53.3%	13.2%

## Data Availability

Not applicable.
